# Improvement of the diabetic foot upon testosterone administration to hypogonadal men with peripheral arterial disease. Report of three cases

**DOI:** 10.1186/1475-2840-8-19

**Published:** 2009-03-28

**Authors:** Svetlana Kalinchenko, Alexandr Zemlyanoy, Louis J Gooren

**Affiliations:** 1 Russian Research Center for Endocrinology, 117136, 11 D, Ulianova, Moscow, Russia; 2 A.V. Vishnevski Institute of Surgery, 117235, 27 B, Serpuchovskai, Moscow, Russia; 3 Vrije Universiteit medical center, de Boelelaan 117, 1081 HV, Amsterdam, The Netherlands

## Abstract

**Background:**

Lower extremity complications (neuropathy, ulceration, infection, and peripheral arterial disease) are common in diabetes mellitus. There is an inverse relation between plasma testosterone and insulin sensitivity, type 2 diabetes mellitus and HbA1c concentrations.

**Methods:**

We report the beneficial effects of administration of testosterone to three men with a diabetic foot whose serum testosterone was subnormal.

**Results:**

Upon normalization of serum testosterone there was an improvement of hyperglycemia, a decrease of leukocytes and of fibrinogen levels, an increase of antithrombin III activity and of tissue oxygen pressure. The wound showed granulation.

**Conclusion:**

Beneficial effects of administration of testosterone to hypogonadal with a diabetic foot may be due to improved vascularization and to anti-inflammatory action.

## Background

Lower extremity complications are common in patients with diabetes and include neuropathy, ulceration, infection, and peripheral arterial disease. Foot infections represent the single most common cause of hospitalization and lower extremity amputation in persons with diabetes. Foot ulceration as a result of diabetic peripheral sensory neuropathy, rigid osseous deformities and soft-tissue contractures, repetitive trauma from unprotected ambulation, and peripheral vascular disease can all lead to a limb- or even life-threatening infection.

Men with type 2 diabetes have a lower serum testosterone concentration compared to men without a history of diabetes, and there is an inverse association between testosterone levels and HbA1c concentrations[1]. A recent systematic review and meta-analysis of cross-sectional studies indicated that testosterone levels were significantly lower in men with type 2 diabetes[2]. Further, in men with low plasma testosterone the risk of diabetes mellitus is increased[3]. One third to one half of men with type 2 diabetes mellitus are now recognized as testosterone deficient. Emerging evidence suggests that testosterone therapy may be able to reverse some aspects of metabolic syndrome[4].

Further, a low plasma testosterone level appeared to be associated with endothelial dysfunction in men independent of other risk factors, suggesting a protective effect of endogenous testosterone on the endothelium[5]. In addition, serum endogenous androgen concentrations were inversely associated with arterial stiffness in men with type 2 diabetes mellitus[6]. There is an association of type 2 diabetes with low testosterone values, and therefore, the effects of an intervention with testosterone are of considerable interest. In hypogonadal men, the few studies on the effects of testosterone treatment on glycemic control were divergent. One study replacing testosterone in hypogonadal men with type 2 diabetes found no effect on glycemic control[7], however another study analyzing 24 hypogonadal men with type 2 diabetes, of which 10 treated with insulin, found that testosterone replacement therapy improved glycemic control[8] confirming an earlier study[9].

In view of the potential relevance of normalization of plasma testosterone for glycemic control in type 2 diabetes and for vascular function, we undertook a pilot study and administered testosterone to men with a diabetic foot and who were found to have plasma testosterone below the reference range.

## Methods

All patients received information that they received experimental treatment' to which they consented. This pilot study was approved by the institute's ethical review board. Written informed consent was obtained from each of the patients for publication of this Case Report and any accompanying images. A copy of the written consent is available for review by the Editor-in-Chief of this journal.

**Figure 1:**
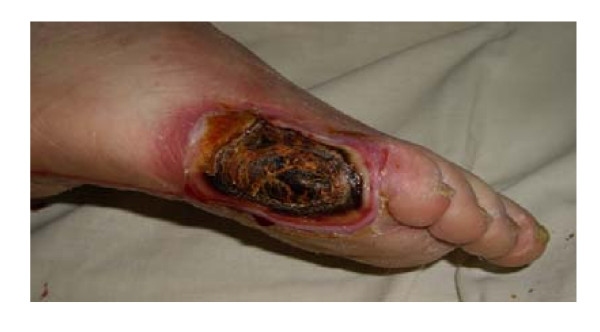
**Patient # 1 before administration of testosterone**.

Patient # 1. A 48-year-old man, presented with diagnosis: type 1 diabetes, dry necrosis of tissues in a projection of V bone of metatarsus (figure 1).

Results of laboratory investigations included hemoglobin (13.1 g/dL), hematocrit (46.6%), leukocytes (9.7 × 10^9^/L), HbA_1c _(7.4%), fibrinogen (6.8 g/L (normal range 2–4 g/L)), antithrombin III activity (87% (normal range 74%–114%)). Other results of blood chemistry were within normal limits, except for plasma glucose (14.0 mmol/L).

Endocrinological findings: total testosterone (7.2 nmol/L (normal range for adult men 12–35 nmol/L)). Bacteriological culture: growth of epidermal staphylococcus in the wound. Using doppler ultrasound severe stenosis of tibial arteries in the distal part of the right anticnemion was found. X-ray examination did not show signs of osteomyelitis of foot bones. Tissue oxygen tension (TpO_2_) was 5 mm Hg. Daily treatment included Cefotaxim 2 g, the heparin Fraxiparin 0.6 ml, Vessel Due F 0.5 LPE, dressing of the wound. In addition, one injection of parenteral testosterone undecanoate (TU) (1000 mg) was administered, whereupon plasma levels of total testosterone rose to 20.3 nmol/L.

**Figure 2:**
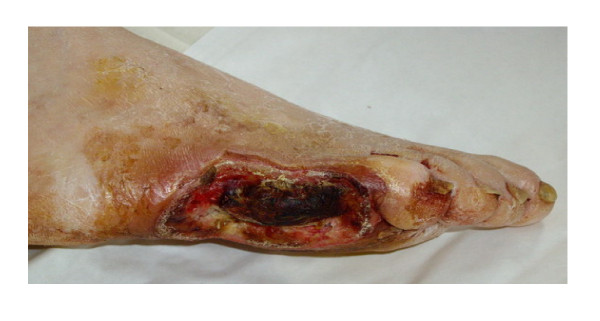
**Patient # 1 after administration of testosterone**.

Results of investigations 17 days after injection of TU: decrease of leukocyte count (5.6 × 10^9^/L), plasma glucose level (6.8 mmol/L), fibrinogen level (5.0 g/L), increased antithrombin III activity (93%), increase of TpO_2 _(18 mm Hg). The wound showed now granulation (figure 2). Plasma glucose level remained stable within the range of 6–8 mmol/L.

**Figure 3:**
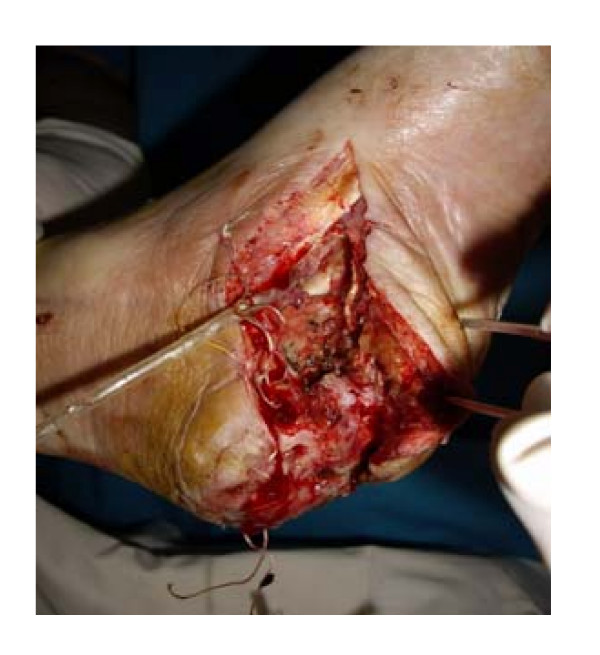
**Patient # 2 before administration of testosterone**.

Patient # 2., a 77-year-old man, presented to our department with diagnosis: type 2 diabetes, phlegmone of the right foot, osteomyelitis of the right calcaneus, critical ischemia of the right leg, coronary heart disease, heart failure, bilateral hydrothorax requiring continuous drainage of pleural effusion amounting to more than 2 litres per day, using permanent vacuum aspiration, renal failure (figure 3).

Results of laboratory investigations while receiving treatment with Netilmicin (0.4 g daily) and fraxiparin (0.6 ml daily): hemoglobin (10.4 g/dL), hematocrit (33.1%), leukocytes (12.2 × 10^9^/L), fibrinogen (5.1 g/L (normal range 2–4 g/L)), antithrombin III activity (74% (normal range 74%–114%)), degree of aggregation – 91.9%, rate of thrombosis – 58%/min. Other blood chemistry tests were within normal limits, with exception of plasma glucose (10 mmol/L). A bacteriological culture revealed bacterial growth in the wound. TpO_2 _of the foot was 2 mmHg. Plasma testosterone levels were below normal (total testosterone 10.5 nmol/L and even more so of bioavailable testosterone: 2.58 nmol/L.

**Figure 4:**
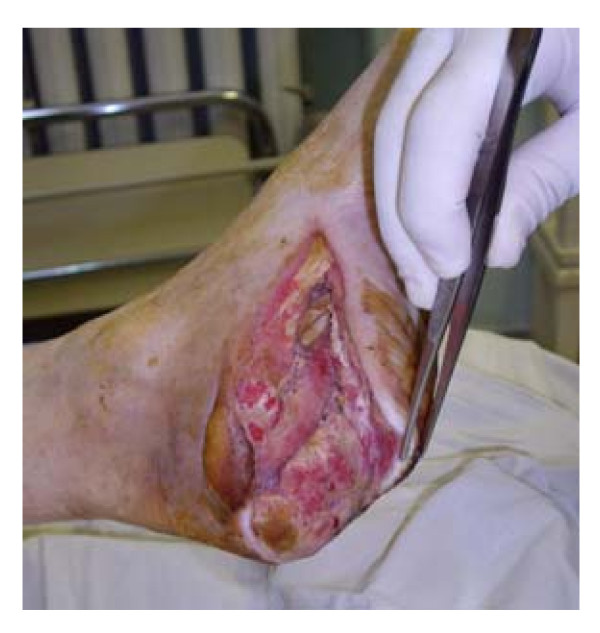
**Patient # 2 after administration of testosterone**.

Results of investigations 25 days after injection of 1000 mg testosterone undecanoate: increase of plasma testosterone to 22.5 nmol/L; stabilization of general condition of the patient, relief of heart failure symptoms, hydrothorax, decrease of leukocyte level (8.0 × 10^9^/L), plasma glucose level (6.5 mmol/L), decrease of a degree of aggregation – 23.8% and rate of thrombosis – 23%/min., increase of antithrombin III activity (88%), increase of TpO_2 _(50 mmHg), increase of the granulation in the wound and occurrence of border epithelization (figure 4). Glucose level remained stable within the range of 5–8 mmol/L.

**Figure 5:**
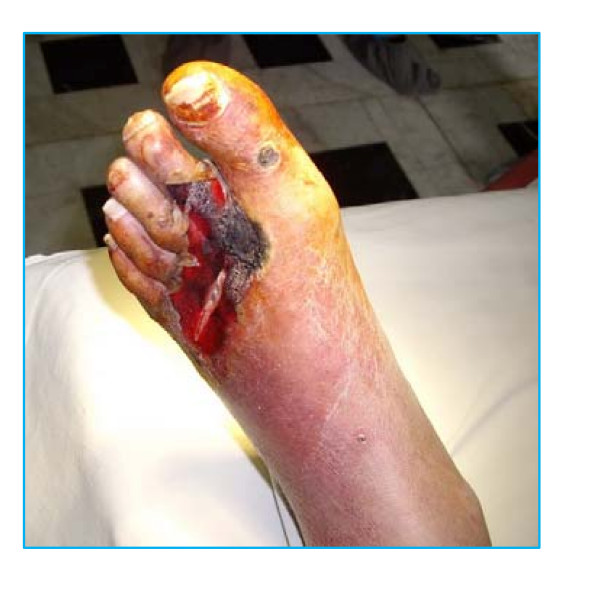
**Patient # 3 before administration of testosterone**.

Patient # 3, a 73 year-old man, presented with the diagnosis: type 2 diabetes, diabetic foot, neuroischemic form. Gangrene of the left foot. Atherosclerotic occlusion of femoropopliteal segment. Critical ischemia of the left foot. Coronary heart disease. (figure 5).

Laboratory investigations: hemoglobin (12.6 g/L), hematocrit (36.4%), leukocytes (12,9 × 10^9^/L), HbA_1c _(13.5%), fibrinogen (6.8 g/L (normal range 2–4 g/L)), antithrombin III activity (63% (normal range 74%–114%)). Other blood chemistry tests were within normal limits, except for plasma glucose level (12.7 mmol/L). Plasma total testosterone was 1.9 nmol/L (normal range for adult men 12–35 nmol/L). Bacteriological culture: growth of epidermal staphylococcus in the wound. Critical stenosis of popliteal artery and arteria tibialis posterior was found by doppler ultrasound of the legs. Tissue oxygen tension (TpO_2_) was 28 mm Hg). The patient underwent superficial femoro-peroneal shunting with autovein and marginal resection with disarticulation of toes 2–5 of the left foot. Daily treatment included Cefotaxim 2 g, Metronidazole 600 mg, Fraxiparin 0.6 ml, Thrombo ASS 100 mg, dressing of the wound. The patient also received one injection of testosterone undecanoate 1000 mg, whereupon plasma levels of total testosterone had risen to 38 nmol/L after 14 days.

**Figure 6:**
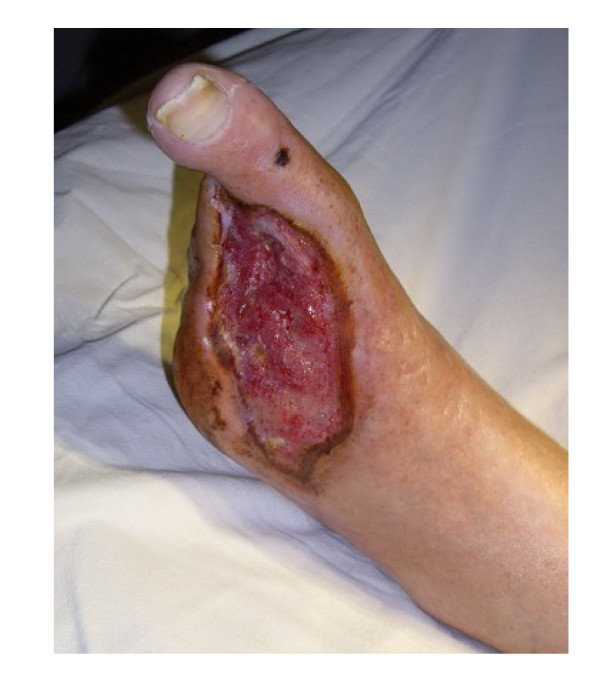
**Patient # 3 after administration of testosterone**.

Test results after 2 months of treatment: decrease of leukocyte level (8,1 × 10^9^/L), plasma glucose level (4.8 mmol/L), fibrinogen level (5.5 g/L), increase of antithrombin III activity (80%), increase of TpO_2 _(37 mm Hg) The wound showed granulation (figure 6). Glucose levels remained stable within the range of 4–7 mmol/L.

## Discussion

There is an abundance of studies establishing that men with the metabolic syndrome, cardiovascular disease and type 2 diabetes have lower-than-normal circulating levels of testosterone. But, as yet, there is a paucity of studies on effects of interventions aiming to restore circulating testosterone to normal[8].

In this pilot study we administered testosterone to men with a diabetic foot who had hypogonadal testosterone levels. There were remarkable improvements.

It is as yet not possible to provide a plausible explanation for these beneficial effects. Recent studies have uncovered a multitude of effects of testosterone in male (patho)physiology which might have played a role.

1) Testosterone has a stimulatory effect on erythropoiesis occuring within weeks after testosterone administration[10]. In our patients, indeed, a rise of hemoglobin levels was found

2) There are many studies now establishing a relationship between low serum testosterone and lower extremity peripheral artery disease[11] but results of intervention studies are few. A recent study found a positive effect of administration of testosterone on vascular function[12] though most earlier studies have reported an impaired flow-mediated vasodilatation upon testosterone administration to hypogonadal men[13]. In vitro-studies or animal studies showed that testosterone can exert acute vaso-relaxing effects via non-genomic mechanisms. These effects involve primarily the vascular smooth muscle, without requiring the presence of endothelium, although an endothelial contribution is apparent in some studies. To date, the mechanism behind the vasodilatory action of testosterone is still under debate and might be through either activation of K (+) channels or blockade of Ca (2+) channels in vascular muscle cells[14].

To explain the beneficial effects of administration of testosterone on the diabetic foot, a parallel may be drawn with erectile dysfunction (ED). ED may originate from vascular insufficiency and the beneficial effects of phospho-diesterase type 5 (PDE) inhibitors is well documented. The vaso-relaxation effect of NO on vascular smooth muscle is mediated by cyclic GMP (cGMP), which is catabolized by phosphodiesterase (PDE). PDE5 inhibitors, which are used to treat erectile dysfunction, increase the bioavailability of cGMP, which activates protein kinase G thereby promoting vasodilatation resulting in penile erection. It is increasingly clear that testosterone plays a role in its own right in the vascular physiology of penile erection promoting NO production[15] and the combination of PDE5 inhibitors with testosterone has been very successful in treating erectile dysfunction[16,17]. In our patients there was an improvement of local oxygen pressures possibly indicating improved vascular function.

3) Apart from effects on the vascular system, testosterone may have had anabolic effects on protein synthesis[18] explaining the beneficial effects of restoring plasma testosterone to the normal range.

4) In animal experimentation androgens at physiological doses inhibit oxidative-stress-induced platelet aggregation via its receptor, which is associated with the reduction of thromboxane A(2) release from platelets[19]. Testosterone has also been found to have a positive effect on fibrinolysis[20]. In our patients there was a decrease of fibrinogen and an increase of antithrombin activity upon testosterone administration.

5) Inflammation is increasingly recognized as an etiological factor in cardiovascular disease [21,22] Testosterone treatment of hypogonadal men shifted the cytokine balance to a state of reduced inflammation[23]. Dihydrotestosterone positively regulated endothelial function through the control of the inflammatory response mediated by nuclear factor-kappaB in endothelial cells[24]. In our study inflammation markers were not studied but there was reduction in leukocyte counts.

6) The actions of 5alpha-dihydrotestosterone and testosterone were primarily deleterious in cutaneous wound healing in a series of studies[25]. This suggests that administration of testosterone might be unfavorable in this regard.

Our findings are observational and should invite studies with an appropriate design to explore whether normalization of testosterone levels in hypogonadal men with a diabetic foot can improve one of the most incapacitating and potentially lethal complications of diabetes mellitus.

## Conclusion

Diabetes mellitus type 2 is very commonly associated with lower-tan-normal serum testosterone levels. In this study three men with a diabetic foot and subnormal serum testosterone, received testosterone treatment. The healing process of the diabetic foot took a favourable turn. This observation invites well-designed studies into the possibly favourable effects of restoring testosterone levels to normal in men suffering from a diabetic foot.

## Competing interests

The authors declare that they have no competing interests.

## Authors' contributions

SK and LG offered the hypothesis that administration of testosterone might be beneficial. The study was carried out by AZ and SK. All three authors have been participating in the interpretation of the data and drafting the contents of the manuscript. Each author has read and approved the final manuscript.
